# Functional Brain Connectivity Patterns of Headache–Mental Disorder Comorbidity in Patients With Migraine

**DOI:** 10.1002/cns.70710

**Published:** 2025-12-26

**Authors:** Tao Yin, Zilei Tian, Lei Lan, Zhengjie Li, Mailan Liu, Yujie Gao, Fanrong Liang, Fang Zeng

**Affiliations:** ^1^ Acupuncture and Tuina School Chengdu University of Traditional Chinese Medicine Chengdu Sichuan China; ^2^ Key Laboratory of Acupuncture for Senile Disease (Chengdu University of TCM) Ministry of Education Chengdu Sichuan China; ^3^ Acupuncture and Brain Science Research Center Chengdu University of Traditional Chinese Medicine Chengdu Sichuan China; ^4^ College of Acupuncture & Moxibustion and Tuina Hunan University of Chinese Medicine Changsha Hunan China; ^5^ Traditional Chinese Medicine School Ningxia Medical University Yinchuan Ningxia China

**Keywords:** anxiety, depression, fMRI, migraine, partial least‐squares correlation, thalamus

## Abstract

**Aims:**

To identify the functional brain connectivity patterns that were correlated with headache and mental conditions in migraineurs and then to elucidate their neurotransmitter basis and explore the potential clinical implications.

**Methods:**

Eighty patients with migraine without aura (MwoA) and 94 healthy controls (HCs) were included. Firstly, we employed partial least–squares correlation (PLSC) to identify a set of resting–state functional connectivity (RSFC) that was co–related with headache symptoms and mental conditions in MwoA patients. Then, we investigated the specific neurotransmitter basis underlying headache–mental disorders‐related RSFC patterns. Finally, we explored the potentials of these RSFC patterns in discriminating patients from HCs, interpreting patient symptoms, stratifying patients into subgroups, and predicting treatment outcomes.

**Results:**

The PLSC analysis revealed one robust latent component linking RSFC between the subcortical nuclei (in particular the thalamus and basal ganglia) and the occipital/temporal cortex to the headache–mental conditions in MwoA patients. These RSFC patterns were spatially correlated with the distribution of several neurotransmitters including 5HT1a, 5HT2a, and mGluR5 receptors. The third part of the analysis indicated that the RSFC patterns could discriminate MwoA patients from HCs with an accuracy of 0.793, differentiate patients into two subtypes, and to some extent predict the efficacy of acupuncture treatment.

**Conclusion:**

This was the first “doubly” multivariate analysis identifying functional brain connectivity patterns underlying headache–mental disorder comorbidity in migraineurs. These findings reveal a neurobiological substrate for migraine–mental disorder comorbidity and highlight the potential of these connectivity patterns as biomarkers for diagnosis and treatment prediction.

## Graphical abstract

This study identified the functional brain connectivity patterns that were correlated with headache and mental conditions in migraineurs and then detected that those features had potential to discriminate patients from healthy controls, stratify patients into subgroups, and predict treatment outcomes.

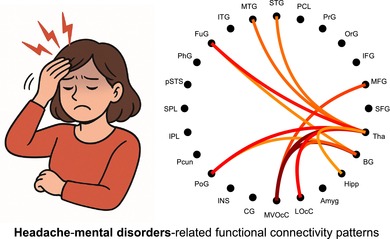

## 1 Introduction

Migraine is a highly prevalent neurological disorder, which affects approximately 14%–15% of the world's population [1], with the highest incidence in young women [2]. Epidemiological study estimates that migraine is now the second most common neurological disorder leading to death and disability, significantly higher than Alzheimer's disease and meningitis [3]. Typical symptoms of migraine include recurrent severe headaches, nausea, vomiting, hypersensitivity to light and sound, and a variety of other physical, mental, and psychological signs and symptoms [4]. These accompanying symptoms, also called comorbidities, greatly increase migraine–related disability and impede the treatment of migraine.

The most widely recognized and intractable comorbidities that affect a wide range of migraineurs are mental disorders, primarily including anxiety and depression [5]. It has been reported that the incidence of migraine in individuals with a history of major depression was three times higher than in those without depression [6]. Similarly, the incidence of depression in individuals with pre–existing migraine was more than 3.7 times higher than in those without a history of migraine [7]. Moreover, genetic analyses indicated that migraine and mental disorders might share the common pathological basis and have bidirectional impacts on each other [8, 9]. On the one hand, migraine and mental disorders, such as depression, have been observed to co–aggregate within families [10] and to share specific gene loci [9, 11]. On the other hand, the interaction between headache and mental symptoms exacerbates patients' condition and impedes the efficacy of treatment [12]. Therefore, elucidating the pathological basis of comorbidity of headache and mental symptoms in migraineurs is of particular urgency, especially for the development of effective pharmacological or non–pharmacological treatments to alleviate the suffering of migraineurs.

Neuroimaging technologies, particularly magnetic resonance imaging (MRI), have emerged as valuable tools for characterizing the central pathology of migraine and provided crucial insights into the comorbidity of headache and mental symptoms in migraineurs [13]. For example, a structural MRI study revealed that patients with both migraine and major depression had reduced brain tissue volume compared with those with either one or neither condition [14]. The functional MRI (fMRI) studies detected that patients suffering from both migraine and depression exhibited diminished activity in the thalamus^15^, heightened activity in the left medial prefrontal cortex (PFC) [15], and reduced resting–state functional connectivity (RSFC) between the amygdala and dorsolateral PFC [16]. Migraineur with anxiety, instead of without anxiety, had higher regional homogeneity in the posterior intraparietal sulcus and higher RSFC between the lingual gyrus and the primary visual cortex [17]. These studies provided valuable first–hand knowledge for understanding headache–mental disorders comorbidity in migraineurs. However, it should be recognized that the group‐level comparisons and univariate statistical analyses (e.g., case–control and independent sample *t*–test) applied in these studies did not provide comprehensive insight into the neuroimaging phenotypes associated with headache–mental disorders comorbidity in migraineurs. These approaches tend to smooth out the distinctive properties of individuals and may overlook the potentially complex associations among different neuroimaging and clinical features. Therefore, the precise mechanisms in which headache, anxiety, and depression interact to precipitate disturbances in brain morphology and function remain largely unresolved.

Partial least‐squares correlation (PLSC) provides a viable approach for overcoming this obstacle. PLSC is a “doubly” multivariate statistical technique to investigate the complex relationships between multiple independent variables and dependent variables. Beyond the variable‐by‐variable analysis of univariate methods, PLSC delivers a comprehensive and multivariate perspective to capture the covarying patterns that better reflect the complex system‐level relationships. Given its outstanding performance in addressing dual multivariate and complex collinear problems, PLSC is a particularly useful tool in neuroimaging studies [18]. It has been successfully employed to identify associations between functional brain metrics and behavioral or cognitive measures [19, 20]. For example, recent studies employed PLSC to elucidate a composite dimension of sleep health covarying with functional brain connectivity patterns of the attentional and thalamic networks [21], as well as several distinct sets of whole‐brain RSFC signatures prominently corresponding to general psychopathology, cognitive dysfunction, and impulsivity [20, 22].

Therefore, in the current study, we employed PLSC to investigate the complex relationships between the whole‐brain RSFC and a set of headache and mental measures in an integrated analysis. This approach was taken to identify the functional brain connectivity patterns for the comorbidity of headache and mental disorders in migraineurs. Based on cumulative research evidence [15, 16, 17], we hypothesized that a network comprising multiple pain and emotion processing brain regions, including the thalamus, anterior cingulate cortex (ACC), and PFC, would be identified as the connectomic basis underlying headache–mental disorders in migraineurs. Moreover, these headache–mental disorders‐related RSFC patterns were associated with specific neurotransmitter systems and demonstrated potential clinical implications in diagnosis and treatment.

## 2 Materials and Methods

### 2.1 Participants

Based on whether headache attack is preceded by transient focal neurological aura symptoms, for example, scintillations and paresthesia, migraine is generally categorized into migraine with aura or migraine without aura (MwoA). The current study focused its analysis on MwoA patients, who represent over 70% of all migraine cases [23].

A total of 86 MwoA patients and 100 HCs were enrolled. These patients were recruited from the outpatient department of the Third Affiliated Hospital of Chengdu University of Traditional Chinese Medicine and were diagnosed by a neurologist in accordance with the International Classification of Headache Disorders for Migraine Without Aura [24]. The HCs were recruited via advertisements in local universities and communities. The inclusion and exclusion criteria for MwoA patients and HCs can be found in the Supporting Information S1 and our recent study [25].

### 2.2 Clinical Measures

In order to evaluate their clinical conditions, the included MwoA patients were required to record headache diaries over a 4‐week observation period and during the 4‐week treatment period [26]. The clinical measures included (1) the duration of illness, (2) the number of days with migraine attacks, which was calculated by summing the numbers of days with migraine attacks in the observation and treatment period, (3) the intensity of headache, which was evaluated with the averaged Visual Analogue Scale (VAS) score of every migraine attack, (4) the duration of migraine attacks, which was measured by the averaged lasting time of every migraine attack, (5) the Role Function‐Restrictive subscale of Migraine‐Specific Quality of Life Questionnaire (MSQ) [27] score, (6) the Role Function‐Preventive subscale of MSQ score, (7) the Emotional Function subscale of MSQ score, (8) the Self‐rating Anxiety Scale (SAS) [28] score, and (9) the Self‐Rating depressive Scale (SDS) [29] score. The aforementioned clinical measures provided a comprehensive evaluation of migraine‐related suffering, including headache symptoms, headache‐related quality of life (QoL), and mental conditions. These clinical measures were evaluated at the baseline as well as after acupuncture treatment.

### 2.3 MRI Data Acquisition and Preprocessing

MRI data of MwoA patients and HCs were acquired with the same 3.0 T Siemens MRI scanner equipped with an eight‐channel phase‐array head coil at the West China Hospital MRI center. The scanning procedure consisted of a high‐resolution three‐dimensional T1‐weighted sequence and a resting‐state blood oxygenation level‐dependent fMRI sequence. The scanning parameters can be found in Supporting Information S2. All participants were instructed to remain awake and keep their heads still throughout the MRI scan, with their eyes closed and ears plugged. In order to minimize the potential impact of acute episodes of headache, patients were required to be migraine‐free for a minimum of 72 h prior to undergoing the MRI scan.

The MRI data was preprocessed with SPM12 (http://www.fil.ion.ucl.ac.uk/spm) and DPARSF 5.2 [30] (http://rfmri.org/DPARSF) toolboxes, based on MATLAB 2017b. The data processing was conducted in accordance with the predefined pipeline of the toolbox. The data preprocessing procedure comprised six steps: (1) discarded the first 10 timepoints allowing for signal stabilization; (2) realigned the time series of images; (3) co‐registered the individual T1‐weighted images to the mean functional images and segmented images to gray matter, white matter, and cerebrospinal fluid; (4) normalized images to the Montreal Neurologic Institute space; (5) regressed out the covariates using the Friston 24 parameter model [31]; and (6) performed temporal band‐pass filtering (0.01–0.08 Hz) and smoothing with a 4 mm Gaussian kernel of full‐width at half maximum across time series.

Given that excessive head motion has significantly impacted the reliability of functional brain network analysis [32], it is necessary to implement strict head motion control criteria. In this study, we applied the mean Jenkinson framewise displacement > 0.2 as the standard for excluding ineligible subjects [33].

### 2.4 RSFC Matrix Construction

The whole‐brain RSFC matrix was constructed based on the Brainnetome Atlas [34], which divided gray matter of the brain into 210 cortical regions and 36 subcortical regions. For each participant, the time course of each node was extracted by calculating the mean value of the voxels. Then, the RSFC of each pair of nodes was measured with *Pearson's* correlation between the time courses of the nodes. A *Fisher's r*‐to‐*z* transformation was performed to improve the normality of the correlation coefficients, resulting in a 246 × 246 RSFC matrix for each participant.

### 2.5 Partial Least‐Squares Correlation Analysis

We used PLSC analysis to examine the relationship between functional brain connectivity patterns and headache and mental disorders‐related measures in MwoA patients. PLSC analysis derived latent components (LCs) by finding weighted patterns of variables from two given matrices that maximally covary with each other [21, 35]. In the present analysis, the first variable set corresponded to the RSFC matrix and the other to the nine clinical measures. Each LC was characterized by a distinct RSFC pattern (called RSFC saliences) and a distinct clinical profile (called clinical saliences), reflecting the contribution of the original variables to the LCs. By linearly projecting the RSFC and clinical measures of each participant onto their respective saliences, we obtained individual‐specific RSFC and clinical composite scores for each LC. To interpret which RSFC and clinical measures drive these LCs, we computed *Pearson's* correlations (called loadings) between the original RSFC and RSFC composite scores and between the original clinical measures and clinical composite scores for each LC. A large loading for a particular clinical measure for a given LC indicated greater importance of the clinical measure for the LC. Similarly, a large loading for a particular RSFC for a given LC indicated greater importance of the RSFC for the LC. Mathematical details of the analysis are described in the manual of myPLS toolbox [36] (https://github.com/danizoeller/myPLS).

The number of significant LCs was determined by a permutation test (1000 permutations). The significance of the RSFC loading and clinical loading was assessed by bootstrap estimation (1000 bootstrapping). *z*‐scores were calculated by dividing each correlation coefficient by its bootstrap‐estimated standard deviation and then converted to *p*‐values. *p*‐values of the permutation test and bootstrap estimation were corrected for multiple comparisons with False Discovery Rate (FDR) *p* < 0.05 based on the *fdr_bh* Function in MATLAB.

To assess the consistency of the results across brain atlases of varying granularity, we further calculated the gyrus‐level and lobe‐level RSFC loading matrix by averaging the significant RSFC loadings within and between 24 gyri and 7 lobes. The 24 gyri and 7 lobes were defined by the Brainnetome Atlas [34].

### 2.6 Spatial Correlation With Neurotransmitter Densities

To investigate the spatial metabolic basis of the functional brain connectivity patterns associated with headache and mental disorders comorbidity in migraineurs that were detected in PLSC analyses, we performed the spatial correlation analyses between RSFC loadings and densities of several neurotransmitters. According to a recent comprehensive review [8], serotonin receptors (5‐HT1a, 5‐HT1b, and 5‐HT2a), metabotropic glutamate receptor 5 (mGluR5), N‐methyl‐D‐aspartate (NMDA) receptor, and γ‐Aminobutyric acid type a (GABAa) receptor play important roles in the onset and clinical progress of migraine and its mental comorbidities, such as anxiety and depression. Therefore, referring to Hansen J and colleagues' study [37], we obtained the averaged group spatial distribution maps of these 6 neurotransmitters from 185 healthy subjects (5‐HT1a: *n* = 36; 5‐HT1b: *n* = 22; 5‐HT2a: *n* = 19; mGluR5: *n* = 73; NMDA: *n* = 29; GABAa: *n* = 6). These maps were then resampled to an isotropic 3 mm spatial resolution to fit our fMRI data. The resampled neurotransmitter density maps were divided into 246 regions based on the Brainnetome Atlas [34], and the average value of each region was calculated to generate a 6 × 246 matrix. After converting the RSFC loadings to absolute values, the RSFC loadings were summed for each region to represent regions' importance scores, generating a 1 × 246 matrix. Finally, *Pearson's* correlation analyses were performed between the importance scores and the neurotransmitter densities of these regions [38]. The significance of correlation analyses was set to FDR corrected *p* < 0.05.

### 2.7 The Clinical Implications of the Headache and Mental Disorders‐Related Functional Brain Connectivity Patterns

To further understand the potential clinical implications of the headache and mental disorders‐related functional brain connectivity patterns identified in the PLSC analyses, we did the following exploratory analysis: (1) the support vector classification (SVC) analysis was performed to explore the accuracy of discriminating between MwoA patients and HCs based on these RSFC features; (2) the support vector regression (SVR) analysis was applied to explore the reliability of interpreting individual clinical measures of MwoA patients based on these RSFC features; (3) the k‐means clustering analysis was conducted to explore the feasibility of classifying MwoA patients into multiple subtypes based on these RSFC features, and then differences between subtypes of MwoA patients were compared in terms of the baseline clinical symptoms and the improvements of symptoms after acupuncture treatment (the rationale and intervention protocols of acupuncture treatment for migraine were described in the Supporting Information S3 and Figure S1); and (4) the SVR analysis was conducted to explore the reliability of predicting improvements of clinical symptoms after acupuncture treatment based on these RSFC features. See Supporting Information S4 for details of this part of analysis.

**FIGURE 1:**
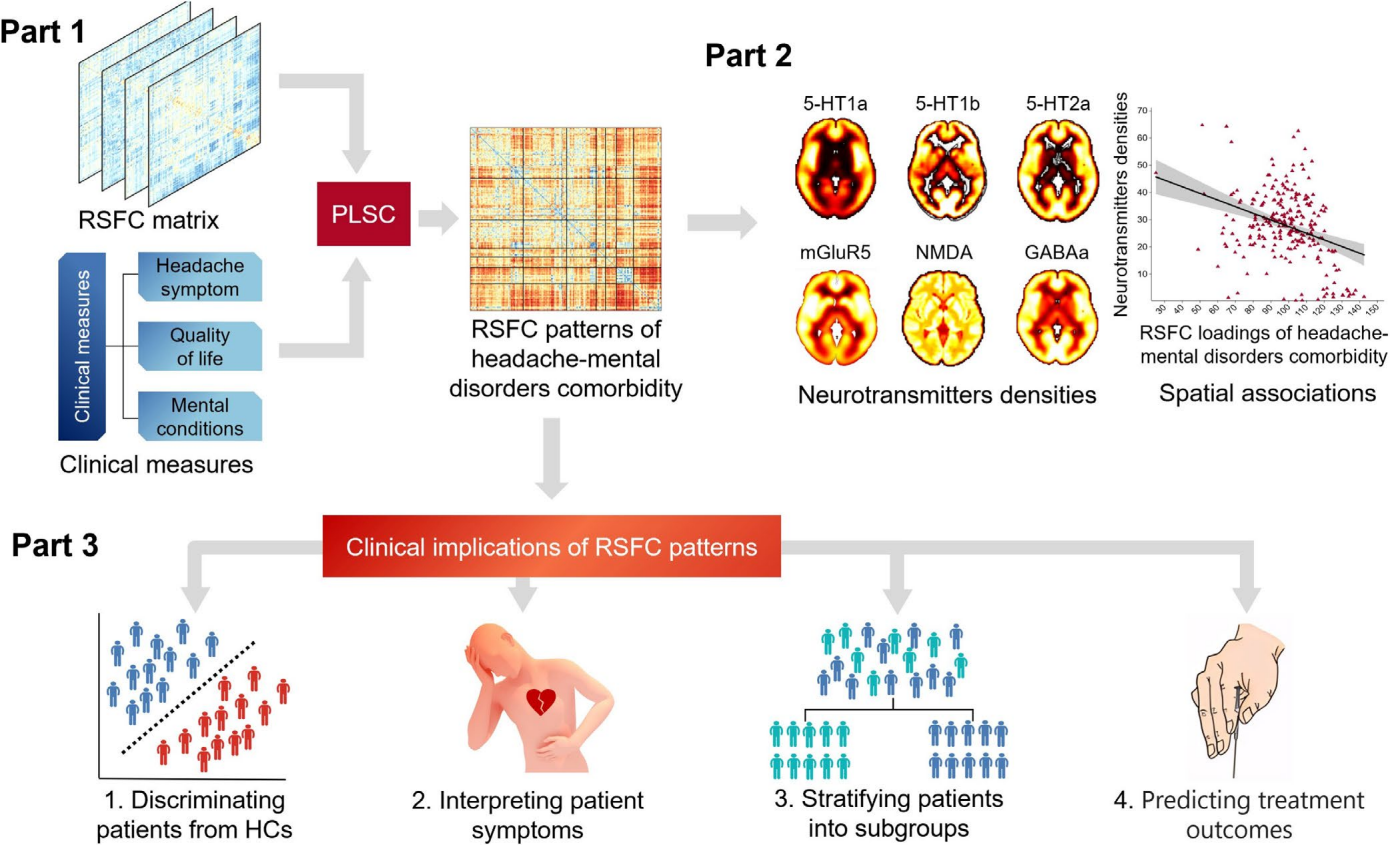
Overview of data analysis processes. 5‐HT1a = 5‐hydroxytryptamine 1a receptor; 5‐HT1b, 5‐hydroxytryptamine 1b receptor; 5‐HT2a, 5‐hydroxytryptamine 2a receptor; GABAa, γ‐aminobutyric acid type a receptor; HCs, healthy controls; mGluR5, metabotropic glutamate receptor 5; NMDA, *N*‐methyl‐D‐aspartate receptor; PLSC, partial least‐squares correlation; RSFC, resting‐state functional connectivity.

Overview of the analysis processes is shown in Figure 1.

## 3 Results

Six MwoA patients and six HCs were excluded for excessive head motion. Therefore, 80 MwoA patients and 94 HCs were included in data analysis. The demographic characteristics and baseline clinical symptoms of MwoA patients and HCs are displayed in Table 1.

**TABLE 1:** Demographic and baseline clinical characteristics of MwoA patients and HCs.

	MwoA (*N* = 80)	HCs (*N* = 94)	Statistics	*p*
Gender (Male/Female)	17/63	31/63	*χ* ^*2*^ = 2.976	0.084
Age (Year)	21.96 ± 2.20	21.52 ± 2.75	*t* = 1.16	0.931
Head motion (Mean framewise displacement)	0.067 ± 0.033	0.068 ± 0.031	*t =* −0.241	0.810
Duration of illness (Months)	65.91 ± 35.83	/	/	/
Number of days with migraine attacks	5.53 ± 4.68	/	/	/
Headache intensity	5.74 ± 1.24	/	/	/
Duration of headache attacks (Hours)	8.39 ± 8.81	/	/	/
Role Function‐Restrictive of MSQ	57.46 ± 17.44	/	/	/
Role Function‐Preventive of MSQ	65.38 ± 20.56	/	/	/
Emotional Function of MSQ	68.23 ± 19.08	/	/	/
SAS	45.18 ± 8.97	/	/	/
SDS	45.39 ± 10.78	/	/	/

Abbreviations: HCs, healthy controls; MSQ, Quality of Life Questionnaire; MwoA, migraine without aura; SAS, Self‐rating Anxiety Scale; SDS, Self‐Rating depressive Scale.

### 3.1 PLSC Analysis Revealed One Robust LC Linking Functional Brain Connectivity Patterns to Headache–Mental Conditions in MwoA Patients

**FIGURE 2:**
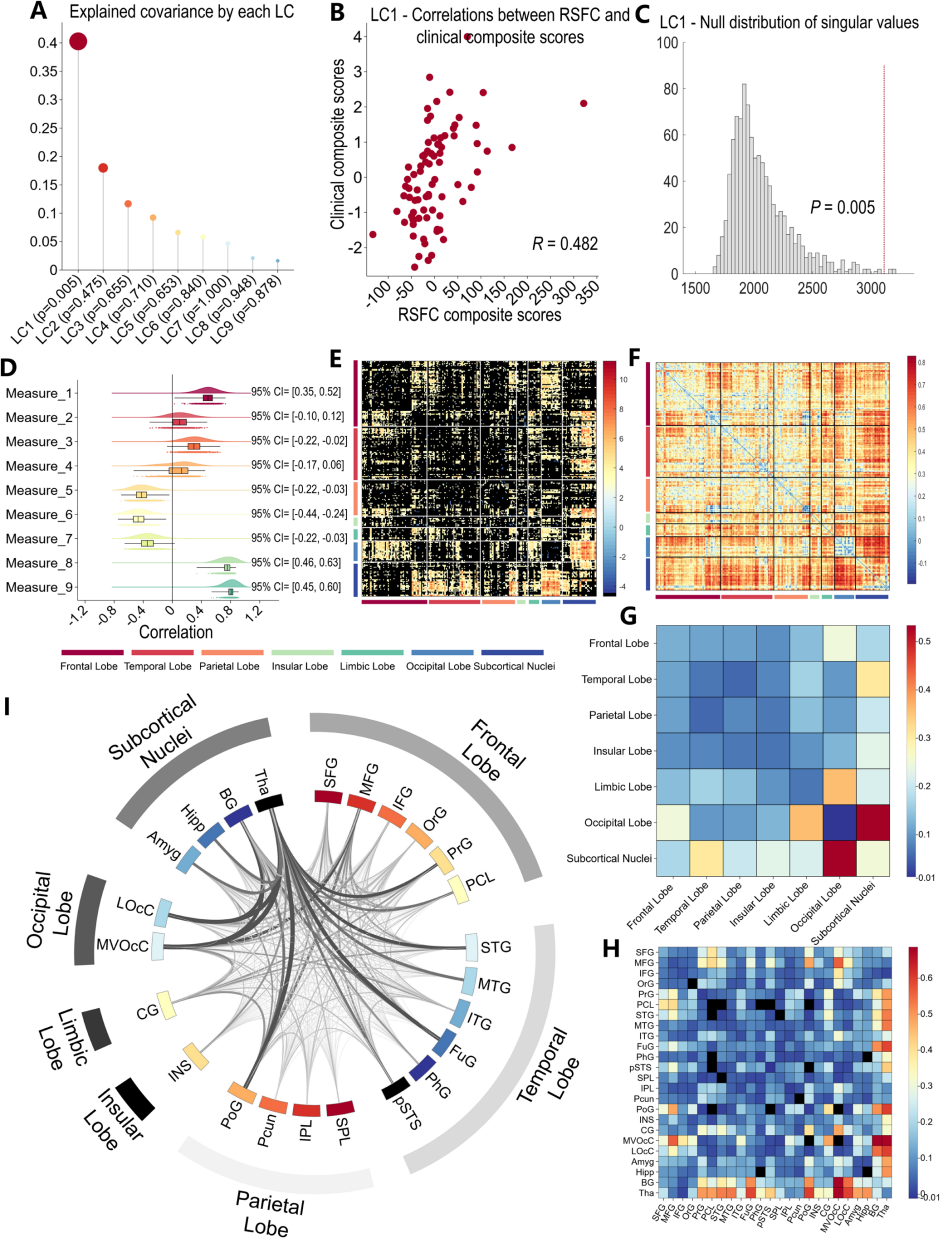
PLSC analysis revealed one robust dimension linking functional brain connectivity pattern to headache and mental conditions in MwoA patients. (A) The amount of covariance explained by each LC. Each dot represents an LC, only the first LC (LC1) survived in permutation tests with FDR correction (*p* < 0.05). (B) scatter plot to illustrate the significant association between individual‐specific RSFC and clinical composite scores in LC1. (C) The results of 1000 permutation for LC1 (two‐sided, permuted *p* = 0.005). (D) Correlations between clinical measures and their corresponding composite scores (clinical loadings). Points under the boxes indicate bootstrapped standard deviation with 1000 bootstrap estimations. There were 7 of 9 clinical measures that showed significant correlations with their composite scores (marked with asterisks). Measure 1–9 indicates duration of illness, number of days with migraine attacks, headache intensity, duration of headache attacks, role function‐restrictive of MSQ, role function‐preventive of MSQ, emotional function of MSQ, SAS, and SDS, respectively. (E) Thresholded (FDR *p* < 0.05) correlations between RSFC and their composite scores (RSFC loadings). (F) Unthresholded correlations between RSFC and their composite scores. (G) lobe‐level correlations between RSFC data and their RSFC composite scores, averaged within and between the 7 lobes defined by the Brainnetome Atlas (lobe‐level RSFC loadings). (H) gyrus‐level correlations between RSFC data and their RSFC composite scores, averaged within and between the 24 gyri defined by the Brainnetome Atlas (gyrus‐level RSFC loadings). (I) Circos plot represents the gyrus‐level RSFC loadings. The outer layer of the circle represents different lobes, and the inter layer represents the different gyri. The thicker and darker the edges between the two gyri, the greater loading of this edge. Abbreviations: The abbreviations of the brain gyri can be found on the website of Brainnetome Atlas: https://atlas.brainnetome.orG. LC = latent components; RSFC = resting‐state functional connectivity.

The PLSC analysis was performed to link the 30,135 (246 × (246–1)/2) RSFC features to nine clinical measures. Figure 2A shows the amount of covariance explained by each LC. Only one LC (LC1) survived in permutation testing with FDR *p* < 0.05. This LC accounted for 40.29% of the RSFC‐clinical measures covariance, with a significant association between RSFC and clinical composite scores (*R* = 0.482, permuted *p* = 0.005) (Figure 2B,C).

Figure 2D shows loadings of the clinical composite scores. Of these, 7 clinical measures survived in the bootstrapping estimation (FDR *p* < 0.05). The higher clinical loadings were associated with longer duration of illness, worse headache and mental disorders (higher VAS, SAS, and SDS scores), and poorer QoL (lower MSQ scores). The loadings of the RSFC composite scores are shown in Figure 2E (without threshold) and Figure 2F (with threshold). The greater RSFC loadings were associated with increased RSFC mainly between the regions of subcortical regions and regions of the occipital and temporal lobes.

Figure 2G–I display the RSFC loadings averaged among the 7 lobes and 24 gyri, with the aim of presenting RSFC loadings on a larger spatial scale. As shown in Figure 2G, the lobe‐level RSFC loadings were mainly involved in higher RSFC between the subcortical regions and occipital lobe. As shown in Figure 2H,I, the gyrus‐level RSFC loadings were concerned with the higher RSFC between the thalamus and the medio‐ventral occipital cortex (MVOcC), lateral occipital cortex (LOcC), postcentral gyrus (PoG), precentral gyrus (PrG), paracentral lobule (PCL), superior and middle temporal gyrus (STG, MTG), fusiform gyrus (FuG), amygdala and hippocampus, as well as higher RSFC between the basal ganglia and the MVOcC, LOcC, PoG, and FuG, between the middle frontal gyrus (MFG) and the MVOcC, PoG.

### 3.2 Spatial Correlation Between Headache and Mental Disorders‐Related Functional Brain Connectivity Patterns and Neurotransmitter Densities

**FIGURE 3:**
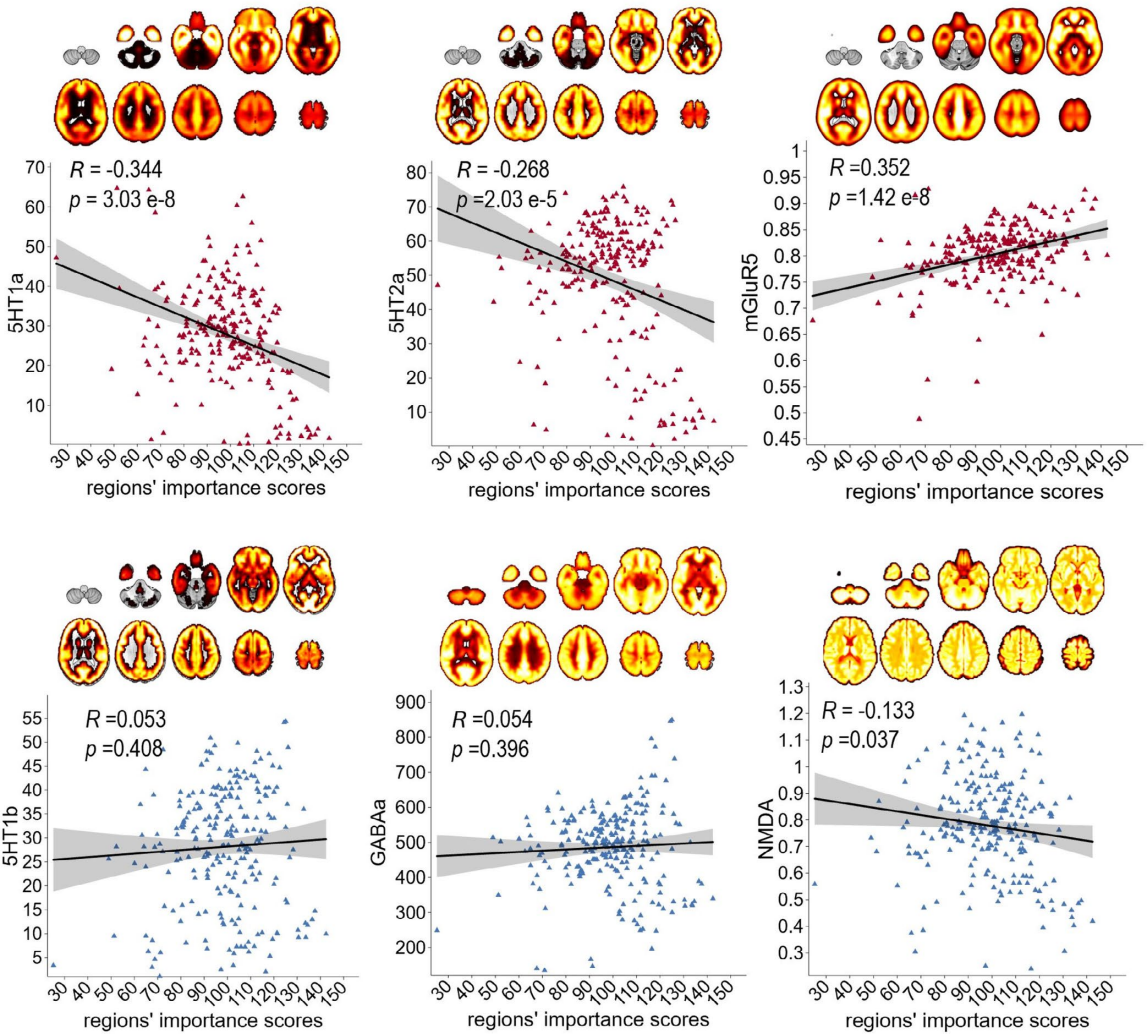
The spatial correlation between headache and mental disorders‐related functional brain connectivity patterns and neurotransmitter densities. Regions' importance scores were generated by summing the absolute RSFC loadings for each of the regions. 5‐HT1a, 5‐hydroxytryptamine 1a receptor; 5‐HT1b, 5‐hydroxytryptamine 1b receptor; 5‐HT2a, 5‐hydroxytryptamine 2a receptor; GABAa, γ‐Aminobutyric acid type a receptor; mGluR5, metabotropic glutamate receptor 5; NMDA, *N*‐methyl‐D‐aspartate receptor.

To test the spatial correlation between the RSFC loadings and the distribution of several neurotransmitters co‐expressed in headache and mental disorders, we conducted the second part of analysis. As shown in Figure 3, there were significant associations between regions' importance scores and the spatial distribution of the 5HT1a, 5HT2a, and mGluR5 receptors (FDR correction, *p* < 0.05). These results suggested that the RSFC patterns, which were significantly associated with headache–mental disorders comorbidity, could be interpreted from the neuromolecular perspective.

### 3.3 The Clinical Implications of Headache and Mental Disorders‐Related Functional Brain Connectivity Patterns

**FIGURE 4:**
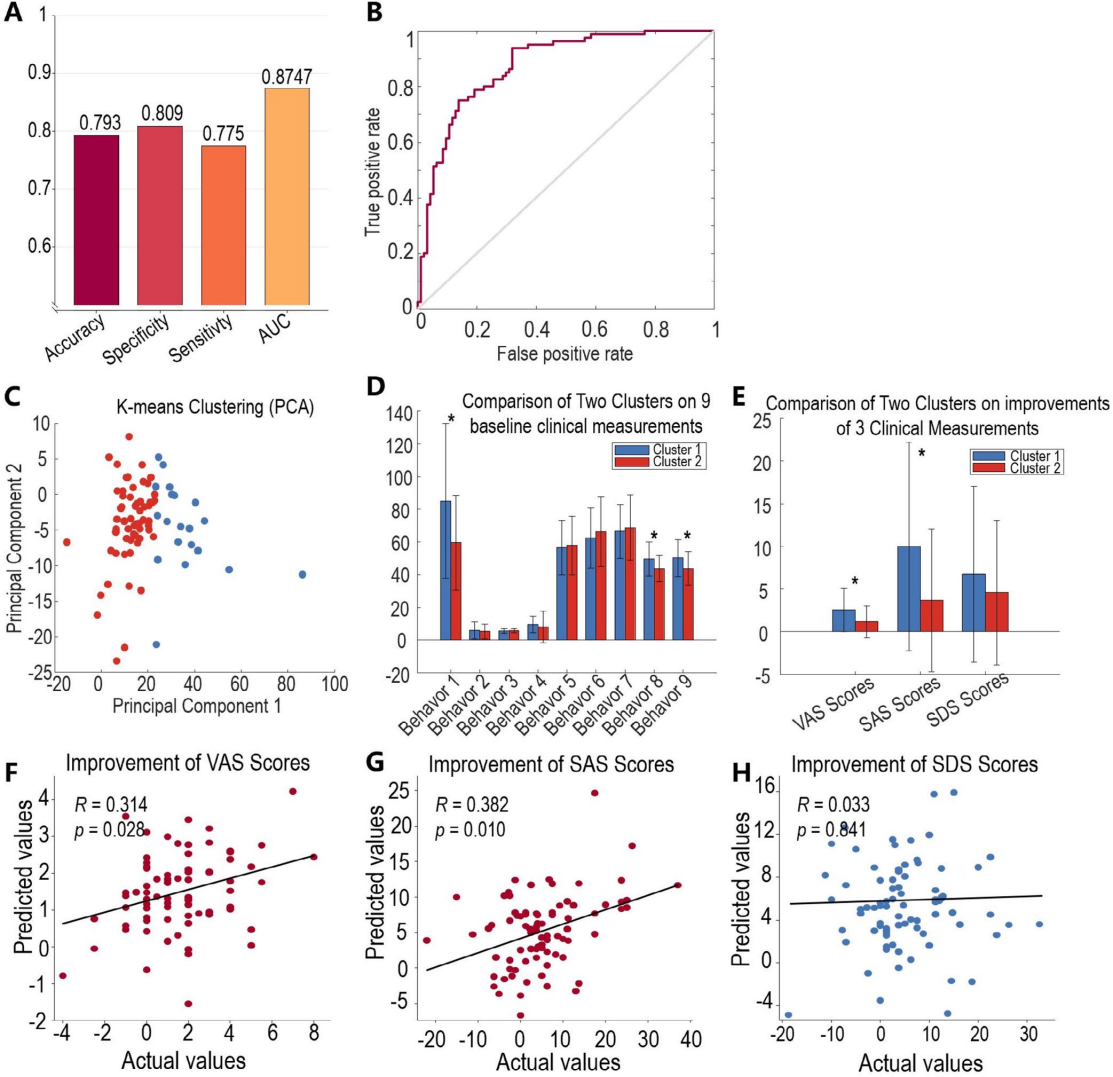
The clinical implications of the headache and mental disorders‐related RSFC patterns. (A, B) The implication of these RSFC in discriminating MwoA patients from HCs. (C–E) The implication of these RSFC in stratifying patients into subgroups. Behavor 1–9 indicates the 9 clinical measures. (F–H) The implication of these RSFC in predicting treatment outcomes. Abbreviations: AUC, area under ROC curve; PCA, principal component analysis; VAS, Visual Analogue Scale; SAS, Self‐rating Anxiety Scale; SDS, Self‐Rating depressive Scale.

To further explore the potential clinical implications of functional brain connectivity patterns associated with headache–mental disorders comorbidity in MwoA patients, we performed the third part of analysis. The first analysis showed that the SVC classifier, which was developed based on the headache–mental disorder‐related RSFC features, could discriminate MwoA patients from HCs with an accuracy of 0.793 (permuted *p* = 0.003), sensitivity of 0.775, specificity of 0.809, and AUC of 0.875 (permuted *p* = 0.001) (Figure 4A,B).

Secondly, nine SVR models were developed to explore the reliability of interpreting each clinical measure based on these RSFC features. The results showed that the functional brain connectivity patterns could not interpret any of the clinical measures, not only the headache‐related but also the mental disorder‐related measures. This result suggested that these RSFC patterns comprehensively represented the overall condition rather than a single symptom of migraineurs. See Supporting Information S5 and Figure S2 for detailed results of these analyses.

Thirdly, k‐means clustering analysis was performed to stratify MwoA patients into several subtypes based on these RSFC features. Determined by the elbow criterion, we identified two cluster centroids and assigned the 80 MwoA patients to the two subgroups, with 20 in cluster 1 and 60 in cluster 2 (Figure 4C). The average silhouette coefficient is 0.328, indicating moderate robustness of the clustering results. There were differences not only in baseline conditions but also in improvements in clinical symptoms between the two clusters of patients (Table 2). Specifically, patients in cluster 1 had longer illness duration and higher SAS and SDS scores (Figure 4D), as well as greater improvement in symptoms, especially in VAS score and SAS score (Figure 4E), than patients in cluster 2 (FDR correction, *p* < 0.05).

**TABLE 2:** Between‐group differences in baseline symptoms and symptom improvements in two clusters of MwoA patients.

	Cluster1 (*N* = 20)	Cluster2 (*N* = 60)	Statistics	*p*
Duration of illness (Months)	85.05 ± 47.22	59.53 ± 28.90	2.883	0.005**
Number of days with migraine attacks	6.00 ± 5.25	5.37 ± 4.52	0.521	0.604
Headache intensity	5.55 ± 1.40	5.81 ± 1.18	−0.807	0.422
Duration of headache attacks(hours)	9.58 ± 4.98	7.99 ± 9.76	0.694	0.490
Role Function‐Restrictive of MSQ	56.56 ± 16.49	57.76 ± 17.87	−0.266	0.791
Role Function‐Preventive of MSQ	62.48 ± 18.37	66.34 ± 21.29	−0.726	0.470
Emotional Function of MSQ	66.46 ± 16.36	68.83 ± 19.99	−0.478	0.634
SAS	49.61 ± 10.50	43.71 ± 7.95	2.646	0.010**
SDS	50.16 ± 11.36	43.80 ± 10.18	2.352	0.021*
Improvement in Headache intensity	2.55 ± 2.52	1.18 ± 1.85	2.622	0.011*
Improvement in SAS	9.99 ± 12.21	3.69 ± 8.37	2.582	0.012*
Improvement in SDS	6.75 ± 10.30	4.60 ± 8.48	0.928	0.356

Abbreviations: HCs, healthy controls; MSQ, quality of life questionnaire; MwoA, migraine without aura; SAS, Self‐rating Anxiety Scale; SDS, Self‐Rating depressive Scale. **p < 0.01; *p < 0.05.

Finally, the SVR algorithm was applied to explore the feasibility of predicting symptom improvement after acupuncture treatment based on these RSFC features. The results demonstrated that it was possible to predict the improvement in clinical symptoms in MwoA patients based on these headache and mental disorders‐related RSFC features (with small but significant predictive value). Specifically, these prediction models achieved an R of 0.314 (*p* = 0.028), mean squared error (MSE) of 4.087 (*p* = 0.014) in predicting VAS score improvements (Figure 4F), an R of 0.382 (*p* = 0.010), MSE of 82.856 (*p* = 0.006) in predicting SAS score improvements (Figure 4G), and an R of 0.033 (*p* = 0.841), MSE of 93.077 (*p* = 0.403) in predicting SDS score improvements (Figure 4H).

Taking together, these results suggested a potential role for these headache–mental disorders‐related functional brain connectivity patterns in identifying and stratifying MwoA patients, as well as predicting their clinical outcomes.

## 4 Discussion

Although it is widely recognized that migraineurs are frequently comorbid with anxiety and depression [39, 40], the underlying neurobiological mechanism behind the co‐occurrence and interaction of headache and emotional disorders has yet been thoroughly elucidated. This study used PLSC, a data‐driven multivariate method, to identify a set of functional brain connectivity patterns significantly associated with headache symptoms and emotional conditions in MwoA patients. In additional analyses, we further investigated the neurotransmitter basis underlying these functional brain connectivity patterns and explored their potential clinical applications. Our study revealed that RSFC between the subcortical nuclei (in particular the thalamus and basal ganglia) and the occipital and temporal cortex largely explained the comorbidity of headache and emotional disorders in MwoA patients. Moreover, the RSFC patterns were spatially correlated with the distribution of several neurotransmitters involved in both migraine and mental disorders, which provided neuroendocrine insights into migraine–mental disorders comorbidity. Exploratory analyses indicated that the RSFC patterns could discriminate MwoA patients from HCs, differentiate patients into two subtypes, and to some extent predict the efficacy of acupuncture treatment. Taking together, these results delineated the RSFC‐guided dimension of headache–mental disorders comorbidity, which could serve as a basis for the development of reliable brain connectome biomarkers and neuromodulation methods with potential for clinical translation, and ultimately for the diagnosis, prognosis, and treatment of migraine‐related problems.

As shown in Figure 2, seven out of the nine clinical measures were found to contribute to the construction of LC1. The disease duration, headache intensity, anxiety, and depression scores made positive contributions to this LC, whereas the three QoL scores made negative contributions. Among these, the anxiety and depression scores contributed most to the construction of this LC. This finding suggested that the RSFC patterns identified by the PLSC method comprehensively represented the overall clinical condition of MwoA patients, which was characterized by more severe headache and mental disorders, longer disease duration, and lower QoL. Due to methodological limitations, previous studies have almost focused on the associations between one brain and one behavioral variable (univariate) or multiple variables against one brain/behavior feature (“single” multivariate), to reveal the functional and structural brain alterations associated with clinical symptoms in migraineurs. Although these studies have contributed to our understanding of the pathological mechanisms of migraine, they invariably have a major limitation: reflecting a single clinical feature, such as pain intensity, disease duration, or anxiety state, instead of a comprehensive representation of the neuropathological patterns of diverse clinical symptoms. Our study applied PLSC, the “doubly” multivariate approach, to address this limitation. This method parsed the multifaceted nature of both brain and behavior simultaneously and provided comprehensive RSFC representations reflecting the underlying patterns of headache and emotional disorders in migraineurs, which have never been reported or mentioned in previous studies. What's even more interesting is that the third part of exploratory analysis indirectly supported this conclusion. In this study, we applied the SVR algorithm to map this set of RSFC to each of the nine clinical symptoms to explore the interpretation of RSFC patterns to a single clinical symptom, but did not obtain any significant findings. This result emphasized an important conceptual finding that the RSFC patterns were integrative biomarkers of overall disease burden rather than predictors of specific, isolated symptoms of migraineurs.

The functional brain connectivity patterns associated with comprehensive clinical symptoms involved RSFC between the subcortical regions, such as the thalamus and basal ganglia, and the occipital cortex, postcentral gyrus, and temporal cortex, as well as between the occipital cortex (especially the MVoCC) and the PFC. Structural and functional abnormalities in the thalamus, basal ganglia, and the occipital and temporal cortices in migraine patients have been widely reported in previous studies [41, 42, 43]. For example, a systematic review involving 24 studies and 748 migraineurs detected functional activity alterations in the thalamus, occipital cortex, temporal cortex, and orbitofrontal cortex in migraineurs. Moreover, the activity intensity of the thalamus was significantly correlated with the VAS score of patients [43]. There is also evidence that the abnormal connectivity patterns between the thalamus, basal ganglia, and occipital and temporal cortices were significantly associated with clinical symptoms such as disease duration, pain intensity, and anxiety–depression states in migraineurs [44, 45, 46]. As found by Wei et al. [45], neural activity in the left occipital cortex was positively correlated with pain scores, and RSFC between the thalamus and right occipital cortex in migraine patients was negatively correlated with their anxiety scores. Changes in these RSFC patterns were often interpreted as an overall functional impairment in the processing and integration of multisensory information in migraine patients. In a recent study [47], Tu et al. constructed an information projection network centered on the thalamus, linking it to the occipital, temporal, and somatosensory cortices, and illustrated that abnormalities in low‐frequency oscillations generated within the thalamocortical network could disrupt and interfere with the information flow between the thalamus and the cortex, thereby affecting the sensory, cognitive, and motor neural processes in migraine patients. An increasing number of studies have demonstrated the involvement of the PFC and ACC in the emotional component of pain and its comorbidity with mental disorders such as anxiety and depression [48, 49, 50]. Recent evidence directly suggested that neurostimulation or interventions targeting these areas could significantly improve patients' pain and mental conditions [51, 52]. Our study found that a combination of RSFC patterns among the thalamus, which is associated with nociceptive perception, the medial occipital cortex, which is associated with multisensory integration of pain, and the PFC and ACC, which are associated with painful emotions, contributed to the explanation of the combined clinical symptoms in migraineurs. That is to say, this network comprising the thalamus, occipital, temporal, and PFC could be conceptualized as a shared pain‐emotion regulatory circuit that integrates multimodal sensory inputs with emotional and cognitive processing. The dysregulation of this circuitry heightens pain perception and impairs emotional control, with the two processes interacting to the co‐occurrence of headache and emotional symptoms in migraine patients.

The neurotransmitter analysis helped us to further clarify the neurophysiological basis of these RSFC patterns. The previous study reported that serotonin and mGluR5 were the neurotransmitters that regulate numerous physiological functions, and their dysregulation was a crucial component of the pathological processes of migraine, depression and anxiety [8]. 5‐HT1a modulates dopamine neurotransmission to regulate mood and pain perception [53], 5‐HT2a regulates vascular tone of the peripheral vasculature, affecting migraine attacks [54], and mGluR5 participates in the central sensitization by regulating the neuronal synaptic plasticity in pain [55]. Activation of serotonin receptors inhibits glutamate release from sensory neurons to reduce pain transmission [56]. The associations between the spatial distribution of these neurotransmitters and the RSFC patterns found in the present study indicated that the comorbidity of migraine and mental disorders was predominantly regulated by serotonin and mGluR5 receptors.

Since brain representations provide a comprehensive picture of a patient's clinical symptoms, what are their potential clinical applications and translational value? The third part of this study gave answers. Our findings indicated that this set of integrated functional brain connectivity features has potential diagnostic value. Without feature selection, these features could discriminate migraine patients from HCs with an accuracy of nearly 80%, significantly outperforming previous studies [57, 58], suggesting that this set of RSFC patterns characterized the profile of migraine patients. In addition, these features were found to have ability to stratify patients into two subtypes, with significant differences in illness duration and emotional conditions, highlighting the potential role of these RSFC features in the fine‐grained diagnosis of migraine patients. The potential treatment implications of these features were shown in two aspects: (1) highlighting differences in treatment outcomes between patients in different subgroups and (2) offering the potential to predict symptom improvements of patients. Generally, patients with milder symptoms should respond better to treatment. However, it was counterintuitive that the current study found migraine patients with longer disease duration and more severe symptoms were more likely to benefit from acupuncture. This finding was strongly aligned with previous acupuncture‐analgesia studies, which demonstrated that patients reporting more severe pain at baseline experienced greater symptoms relief after acupuncture treatment [59, 60]. These studies attributed these effects to regression to the mean or floor effects at baseline. However, in light of our neuroimaging findings (the severity of baseline symptoms was significantly correlated with patients' RSFC abnormalities), we speculated that the worse baseline symptoms might represent a state with greater neuroplastic potential. Consequently, such patients were likely to derive greater benefit from acupuncture treatment, which was considered as a peripheral neuromodulation method. Although previous studies have not identified brain mapping features corresponding to multiple clinical symptoms, anecdotal evidence has helped to clarify the role of these brain network features in explaining the pathological features of migraine and its clinical response to treatment. For example, our previous study found that spontaneous activity patterns of the bilateral middle occipital gyrus could not only discriminate MwoA patients from HCs, but also predict the outcome of acupuncture treatment in patients [61]. Tu and colleagues' work also found that the occipital gyrus‐related network could discriminate migraineurs from HCs, and changes in these biomarkers showed significant correlation with changes in headache frequency in response to treatment [62]. We speculated here that these results were largely attributable to the comprehensive representation and characterization of clinical symptomatology provided by this functional brain connectivity pattern.

There were several limitations of our study. (1) This study used a data‐driven approach to identify a set of functional brain connectivity patterns that were significantly associated with headache symptoms and anxiety–depression scores in MwoA patients, which was regarded as brain connectome phenotypes of headache‐emotional comorbidity in migraine. However, the definition of mental disorders relied solely on self‐report scales (SAS, SDS) without formal diagnosis by a psychiatrist. This may not accurately capture clinical anxiety/depression, potentially diluting the “comorbidity” signal. It is therefore necessary and beneficial to replicate the results of this study in patients with a dual diagnosis of migraine and anxiety/depression. (2) Study has shown that migraine was highly prevalent in young women [2], and therefore the vast majority of our enrolled patients were young women, which inevitably introduced the gender and age bias and limited the generalizability of the findings to male and older migraine populations. Future validation in more demographically diverse cohorts is needed. (3) Due to the limitation of neurotransmitter database, the analysis focuses solely on six neurotransmitters. Future study could address other neurotransmitters, such as calcitonin‐gene‐related peptide and dopamine receptors, and explore their relationship with the comorbidity of migraine and mental disorders, so as to draw an even more complete molecular picture. (4) In the third part of the study, we used a machine learning approach to explore the potential clinical applications of this set of RSFC patterns. However, train and validation within the same dataset may carry the risk of overfitting and inflate confidence in the performance metrics. Therefore, special attention should be paid to the external generalization of these findings in independent cohorts. (5) Patients were randomized into one of three groups and received the acupuncture treatment with different prescriptions. Although these acupuncture prescriptions were proven effective and analogous in relieving migraine symptoms in our previous clinical trials, it also should be regarded as a potential confounding factor.

## 5 Conclusion

In this study, we identified a set of functional brain connectivity patterns characterized by RSFC of subcortical regions‐occipital/temporal cortex, that were significantly associated with headache symptoms and mental condition in migraineurs. Moreover, the subsequent analysis revealed the significant association of these disease‐related functional brain connectivity patterns with serotonin and mGluR5 neurotransmitters as well as their potential clinical value in the fine‐tuned diagnosis of migraine and the individual prediction of treatment outcome. This was the first doubly multivariate analysis to resolve the brain connectome phenotypes underlying headache–mental disorders comorbidity in migraineurs, and provided prerequisites for deepening the neurological mechanisms of migraine and targeting the implementation of neuromodulation, so as to optimize disease diagnosis and treatment and therefore improve the composite symptoms and alleviate the suffering of migraineurs.

## Author Contributions

F.Z., T.Y., and F.L. designed the study. T.Y. analyzed the data. Z.T. repeated and verified the results. L.L., Z.J., M.L., and Y.G. contributed to the participants' recruitment and data collection. T.Y. wrote the initial manuscript. F.Z. reviewed and edited the manuscript. All authors read and approved the final manuscript.

## Funding

This work was supported by the National Science Fund for Distinguished Young Scholars (No. 82225050), the National Natural Science Foundation of China (No. 81503664, 81590951, 82205285), the Sichuan Science and Technology Program (No. 22ZDYF1034), and the Tianfu Qingcheng Program of Sichuan Province.

## Ethics Statement

The study was approved by the Ethics Committee of Hospital of Chengdu University of Traditional Chinese Medicine (No. 2010KL‐004).

## Consent

All participants provided written informed consent prior to study enrollment.

## Conflicts of Interest

The authors declare no conflicts of interest.

## Supporting information


**Data S1:** cns70710‐sup‐0001‐Supinfo.docx

## Data Availability

The main data supporting our findings can be found within the manuscript. The original data used during the current study are available from the corresponding author on reasonable request.
