# Supplementary material for: Functional Brain Connectivity Patterns of Headache–Mental Disorder Comorbidity in Patients With Migraine
Source: CNS Neurosci Ther. 2025 Dec 26;31(12):e70710. doi: 10.1002/cns.70710 (PMC12741590; doi:10.1002/cns.70710)
Supplement: Supplementary file 1 — Data S1: cns70710‐sup‐0001‐Supinfo.docx [file CNS-31-e70710-s001.docx]

**Functional brain connectivity patterns of headache-mental disorders comorbidity in patient with migraine**

**Supplementary material 1:**

**The** **inclusion and exclusion criteria of the MwoA patients and healthy controls**

**MwoA patients**

The inclusion criteria for MwoA patients were as follows: 1) aged between 17 to 45 years old, 2) right-handed, 3) had migraine symptoms more than 6 months, 4) had at least one attack per month in the past 3 months, 5) did not taking any pharmacologic or non-pharmacologic interventions for migraine in the past 3 months.

The exclusion criteria were as follows: 1) were secondary headache caused by traumatic brain injury, hypertension, or any other organic reasons, 2) comorbid any severe primary illnesses, 3) had any other chronic pain conditions, 4) had contraindication of MRI scanning, e.g., claustrophobia.

**Healthy controls**

The inclusion criteria of healthy subjects: 1) aged between 17 to 45 years old, 2) right-handed, 3) were free from any chronic pain disorder.

The exclusion criteria of healthy subjects: 1) had any organic or functional disease during a routine physical examination, 2) had contraindication of MRI scanning, e.g., claustrophobia.

Both MwoA patients and healthy controls underwent comprehensive history taking, physical examination, and routine laboratory examinations. Subjects with abnormal test results were excluded.

**Supplementary material 2:**

**Scanning parameters**

The T1-Weighted images were obtained with the axial fast spoiled gradient recalled sequence. The scanning parameters were as follows: repetition time/echo time = 1900/2.26 ms, slice thickness = 1 mm, field of view = 256×256 mm^2^, and matrix size = 256×256. The BOLD fMRI images were obtained with echo-planar imaging. The scanning parameters were as follows: 30 contiguous slices number = 30, slice thickness = 5 mm, repetition time/echo time = 2000/30 ms, flip angle = 90°, field of view = 240 × 240 mm^2^, data matrix = 64 × 64, total volumes = 180.

**Supplementary material 3:**

Acupuncture is widely used by Chinese and other Oriental populations for analgesia, and has been gradually accepted as a complementary analgesic therapy in the West. Clinical data suggest that acupuncture can be at least as effective as migraine prophylactic drugs with fewer adverse events, and also relieve acute or chronic migraine symptoms. Therefore, acupuncture treatments were applied in relieving the symptoms of migraineurs.

In the treatment period, patients with MwoA received 20 sessions of acupuncture treatment in 4 weeks (five sessions per week). In each session, patients received manual acupuncture treatment with the disposable sterile filiform needles for 30 min with *deqi* sensation. Two licensed acupuncturists with at least 3 years of clinical experience administered all the acupuncture protocols. details of acupoint prescriptions are shown in **Figure S1**. Each patient was randomly assigned to receive acupuncture treatment with one of three specific acupoint prescriptions. The points in these prescriptions were believed to connect to the head via particular meridians (the Shaoyang and Yangming meridians) and all had been proven effective and analogous in relieving migraine symptoms in our previous clinical trials^1,2^.


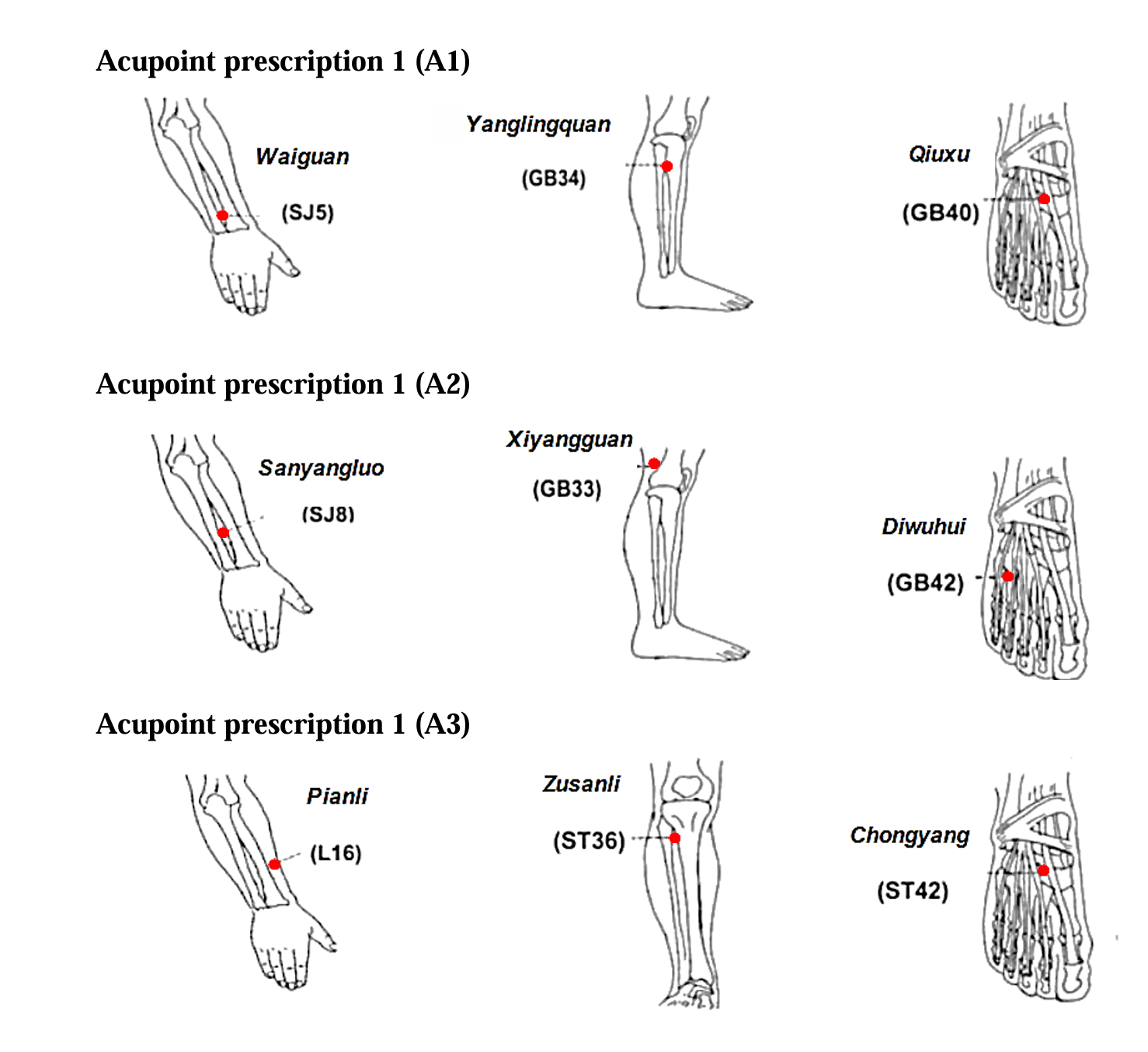


**Figure S1.** Location of acupoints of these three acupoint prescriptions.

**Supplementary material 4:**

**Details of analysis about the** **clinical implications of the headache and mental disorders-related functional brain connectivity patterns**

**Step 1: Discriminating between MwoA patients and HCs based on headache and mental disorders-related functional brain connectivity patterns**

The classification analysis was performed using the LIBSVM toolbox^3^ (https://www.csie.ntu.edu.tw/~cjlin/Libsvm) based on MATLAB 2017b. To test whether these RSFC features could effectively discriminate MwoA patients and HCs, we used the significant RSFC loadings of LCs as the input features and constructed a classification model based on the support vector classification (SVC) algorithm with default parameters (*t* = 0, *c* = 1). The performance of the classifier was evaluated with the 10-fold cross-validation (10-fold CV) strategy. Same as our previous study^4^, the accuracy, sensitivity, specificity, and area under the receiver operating characteristic curve (AUC) were applied to assess the performance of the classifier. The statistical significance of the classification results (accuracy and AUC) was assessed with the permutation test. The number of replicates was set to 1000 (statistical efficiency = 0.001).

**Step 2: Interpretating individual clinical measures of MwoA patients based on headache and mental disorders-related functional brain connectivity patterns**

Similar to the classification analysis, the regression analysis was conducted based on the LIBSVM toolbox and MATLAB 2017b. These features got from the significant RSFC loadings of the LCs were taken as input features, the 9 clinical measures described in the clinical measures section were taken as labels. Nine regression models were constructed based on these features and labels with the default parameter support vector regression (SVR) algorithm (*t* = 2, *c* = 1) to explore the extent to which these features explained the clinical symptoms of patients. Model performance was assessed using the 10-fold CV strategy, with correlation coefficient (R) and mean squared error (MSE) as metrics. Permutation tests were performed with a permutation time of 1000 to assess the statistical significance of R and MSE.

**Step 3: Classifying MwoA patients into multiple subtypes based on headache and mental disorders-related functional brain connectivity patterns**

K-means clustering analysis was performed to classify the MwoA patients with similar RSFC patterns into different subtypes, with the significant RSFC loadings of the LCs as features. K-means clustering is an unsupervised machine learning algorithm whose basic principle is to classify the dataset by the similarity between samples such that the intra-cluster disparity is minimized and the inter-cluster disparity is maximized. The k-means clustering analysis was conducted based on the built-in functions of Matlab 2017b. The similarity between the samples was assessed using the L1 distance. The optimal number of cluster centroids was determined by the elbow criterion, which was defined as the ratio of the within‐cluster distance to the between‐cluster distance^5^. The quality and robustness of the clustering was assessed by silhouette scores.

Subsequently, between-group comparisons were performed to detect possible differences in baseline clinical measures and improvements in clinical symptoms among MwoA patients in different subtypes. Two-sample *t*-tests were used and the threshold for statistical significance was *p* < 0.05, FDR corrected for multiple comparisons.

**Step 4: Predicting clinical improvements of MwoA patients based on headache and mental disorders-related functional brain connectivity patterns**

The prediction models of clinical improvements in MwoA patients were constructed based on the SVR algorithm with default parameters. The significant RSFC loadings of the LCs were taken as input features, and improvements in clinical symptoms compared to baseline were taken as labels. The model construction and evaluation were the same as the analysis in step 2.

**Supplementary material 5:**

**Detailed results of the analysis about interpretating individual clinical measures of MwoA patients based on headache and mental disorders-related functional brain connectivity patterns**

Nine SVR models were construction to interpretate individual clinical measures of MwoA patients based on headache and mental disorders-related functional brain connectivity patterns. As shown in **Figure S2,** none of the nine models performed well in explaining the clinical indicators (permuted *p* for R and MSE both bigger than 0.05).


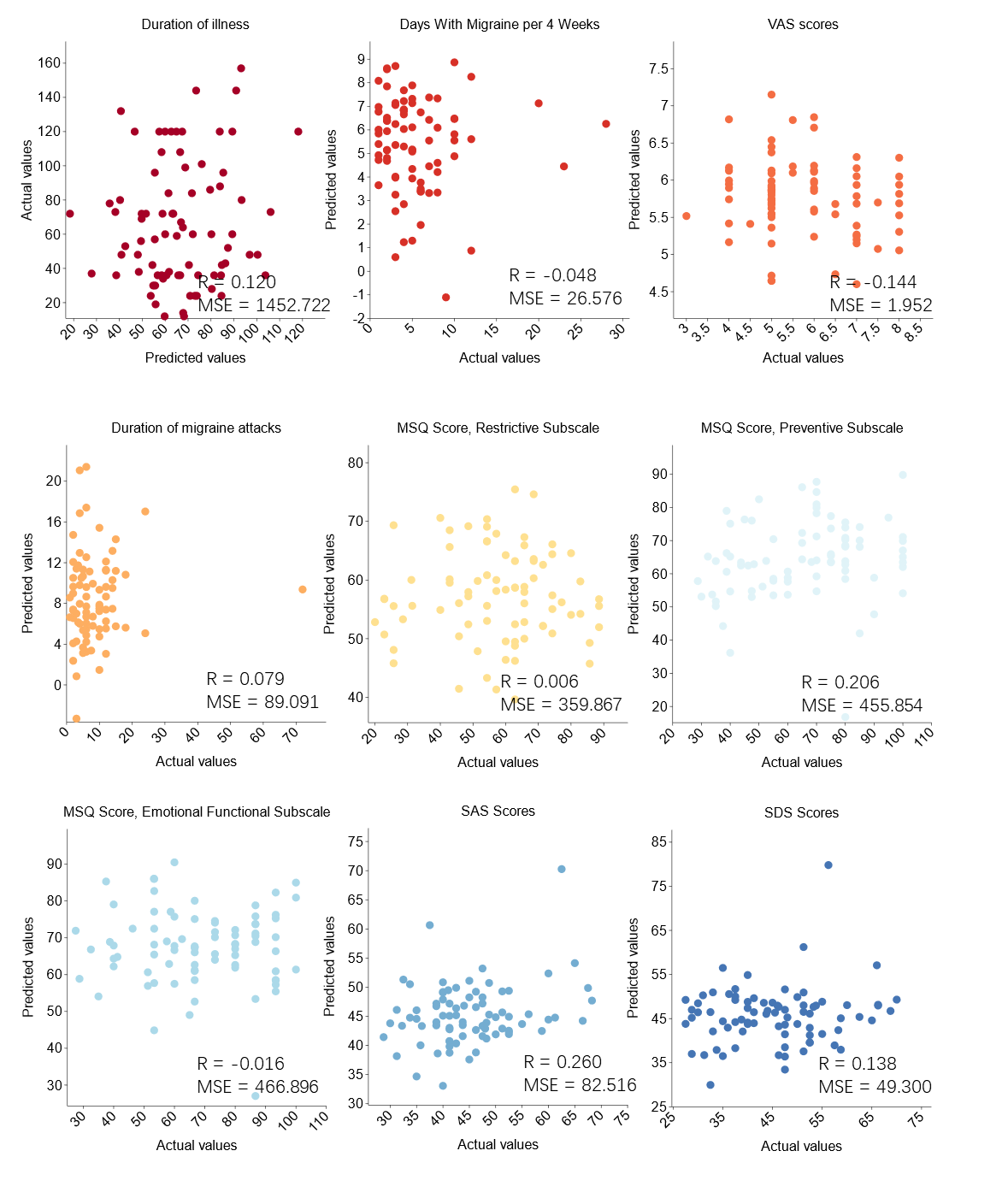


**Figure S2.** The performance of these SVR models in interpreting clinical measures.

**References**

1. Zhao L, Chen J, Li Y, et al. The Long-term Effect of Acupuncture for Migraine Prophylaxis: A Randomized Clinical Trial. *JAMA Intern Med.* 2017;177(4):508-515.

2. Li Z, Lan L, Zeng F, et al. The altered right frontoparietal network functional connectivity in migraine and the modulation effect of treatment. *Cephalalgia.* 2017;37(2):161-176.

3. Chih-Chung C, Chih-Jen L. Libsvm: a library for support vector machines. *ACM Trans Intell Syst Technol, vol 2(3).* 2011:1-27.

4. Yin T, Sun G, Tian Z, et al. The Spontaneous Activity Pattern of the Middle Occipital Gyrus Predicts the Clinical Efficacy of Acupuncture Treatment for Migraine Without Aura. *Front Neurol.* 2020;11:588207.

5. Tu Y, Fu Z, Zeng F, et al. Abnormal thalamocortical network dynamics in migraine. *Neurology.* 2019;92(23):e2706-e2716.
